# Bioresorbable Scaffolds for Below-the-Knee Arterial Disease: A Literature Review of New Developments

**DOI:** 10.31083/j.rcm2504133

**Published:** 2024-04-03

**Authors:** Hong-Jie Cui, Ying-Feng Wu

**Affiliations:** ^1^Department of Vascular Surgery, Xuanwu Hospital, Capital Medical University, 100053 Beijing, China; ^2^Department of Vascular Surgery, Luhe Hospital Affiliated to Capital Medical University, 101199 Beijing, China

**Keywords:** bioresorbable scaffold (BRS), below-the-knee (BTK) disease, chronic limb-threatening ischemia (CLTI), infrapopliteal artery, patency, thrombosis

## Abstract

This review aimed to explore the therapeutic effect of bioabsorbable stents in the inferior genicular artery, from the emergence of absorbable bare metal stents to the latest technology in polymer and anti-proliferative eluting drugs mixed with coated bioresorbable vascular stents (BVSs). Currently, there are conflicting data regarding the safety and effectiveness of BVSs in infrapopliteal artery interventions, especially compared to the current generation of drug-eluting stents (DESs). This review will cover the existing data on BVSs in reconstructing the infrapopliteal arterial blood flow and active clinical trials for future iterations of BVSs. In terms of primary patency rate and target lesion revascularization rate, the available research on the effectiveness of BVSs in reconstructing the infrapopliteal arterial blood flow suggests that a BVS is compatible with current DESs within 3–12 months; long-term data have not yet been reported. The ABSORB BVS is the most studied BVS in cardiovascular disease (CAD). Initially, the ABSORB BVS showed promising results. Managing intricate regions in peripheral artery disorders, such as branching or lengthy lesions, continues to be a formidable undertaking. In contrast to the advanced narrowing of arteries seen in standard permanent stent procedures, bioabsorbable stents have the potential to promote the expansion and beneficial merging of blood channels in the latter stages. Furthermore, incorporating stents and re-establishing the endothelial function can diminish the probability of restenosis or thrombosis. Nevertheless, the extent to which bioabsorbable stents may simultaneously preserve arterial patency and guarantee their structural integrity remains uncertain. The powerful and intricate mechanical stresses exerted by the blood in the superficial femoral artery and popliteal artery can cause negative consequences on any implant inserted into the vessel, regardless of its composition, even metal. Furthermore, incorporating stents is advantageous for treating persistent occlusive lesions since it does not impact later treatments, including corrective bypass operations. Evidence is scarce about the use of bioabsorbable stents in treating infrapopliteal lesions. Utilizing bioabsorbable stents in minor infrapopliteal lesions can successfully maintain the patency of the blood vessel lumen, whereas balloon angioplasty cannot offer this benefit. The primary focus of testing these materials is determining whether bioabsorbable scaffolds can provide adequate radial force in highly calcified elongated lesions. Indeed, using “-limus” medication elution technology in conjunction with bioabsorbable stents has previously offered clinical benefits in treating the popliteal artery, as evidenced by limited trials.BVSs for peripheral arterial disease (PAD) show promise and have the potential to offer a less inflammatory and more vessel-friendly option compared to permanent metallic stents. However, current evidence does not yet allow for a universal recommendation for their use. Thus, ongoing, and future studies, such as those examining the newer generation of bioresorbable scaffolds (BRSs) with improved mechanical properties and resorption profiles, will be crucial in defining the role of BRSs in managing PAD.

## 1. Introduction

Peripheral arterial disease (PAD) is a prevalent and serious disease. It is caused by excessive lipoprotein accumulation in the tunica intima brought on by aberrant lipid metabolism [1, 2]. As a result of damage to the tunica intima, the inner diameter of the arteries may eventually decrease by variable degrees, resulting in limb ischemia. Currently, after a stroke and coronary heart disease, PAD is the third most common manifestation of atherosclerosis, and its prevalence rate increases with age. In 2015, the global prevalence of PAD was approximately 5.6%, which means roughly 300 million people were affected [3, 4]. The prevalence rate in nations around the world is approximately 5% between the ages of 40 and 44 and approximately 12% from 70 to 74 [5]. Men are slightly more likely than women to be over the age of 70 in China, where the prevalence rate ranges from 15% to 20%. Arteriosclerosis obliterans in the lower extremities, which affects almost 70% of symptomatic PAD individuals [6], is characterized largely by infrapopliteal lesions, particularly those in the anterior and posterior tibial arteries [7]. Restoring blood flow in the area below the knee is more difficult than in the iliac artery and femoral and popliteal arteries. Traditional bypass surgery and balloon angioplasty have not been effective in achieving adequate results [8]. When chronic limb-threatening ischemia (CLTI) occurs, characterized by ischemic rest pain, tissue loss, or gangrene, the risk of amputation is higher. Since the infrapopliteal vessels serve as the final distribution points for blood flow to the lower limbs, and there is a direct correlation between the survival of foot tissue and the health of the femoral and popliteal vessels, endovascular treatment of infrapopliteal disease is focused on the treatment of patients with rest pain or critical limb ischemia due to severe atherosclerotic disease [9]. The first-generation devices, which took decades to develop, include bare metal stents (BMSs) and plain old balloon angioplasty (POBA). The second-generation devices, which include drug-coated balloons (DCBs) and drug-eluting stents (DESs), have been used for below-the-knee blood flow reconstruction and have produced specific results. However, third-generation bioresorbable scaffolds (BRSs) have begun to be used in clinical practice due to the limits of the current devices and the concept of the “leave nothing behind” philosophy [10]. However, the superiority of using a BRS remains debatable owing to the lack of large-scale randomized studies.

## 2. Limitations of Previous Devices

All guidelines emphasize the value of endovascular revascularization in establishing blood flow to the foot (IB) [11, 12, 13], in addition to recommendations by the European Vascular Society regarding great saphenous vein bypass surgery as the preferred method (IA) for infrapopliteal revascularization [14].

POBA remains the principal therapeutic approach despite developing several novel devices for restoring popliteal blood flow [15]. The long-term patency rate of POBA surgery is unfavorable because of several problems, including post-dilation dissection [16], elastic recoil [17], residual stenosis [18], and restenosis brought on by endothelial inflammation [19]. While BMSs significantly improve patient amputation-free survival compared to POBA, its secondary intervention rate is nearly twice as high as the one for POBA, meaning a potential increase in medical costs [20].

The use of DESs that constantly release inhibitory medications has become popular in halting the growth brought on by endothelial inflammation and improving patency. Although there is no discernible difference between drug-eluting stents and POBA and BMSs in improving long-term amputation-free survival and mortality in patients, drug-eluting stents can significantly improve primary patency of stents and decrease re-intervention of targeted lesions, according to several meta-analyses containing moderate or low-quality evidence [21, 22, 23]. Drug-eluting stents still have certain mechanical and biological flaws, such as stent fractures, remodeling, and side branch jailing, similar to metal stents [24, 25, 26]. The significant causes of neointima generation with a DES in-stent restenosis also include hypersensitivity to the polymer and the medication, local inflation, delayed heating, and intimal hyperplasia [27, 28], resulting in unsatisfactory long-term results.

DCBs, similar to DESs, exhibit favorable immediate outcomes; however, the mid-term and long-term follow-up results have varied. Jia *et al*. [29] found that primary patency at 6 months was 75.0% in the DCB group and 28.3% in the control group (*p* < 0.001), while late lumen loss was 0.43 ± 0.62 mm for DCBs vs. 0.99 ± 0.55 mm for the controls (*p* < 0.001). Freedom from clinically driven target lesion revascularization (CD-TLR) at 12 months was 91.5% in the DCB group vs. 76.8% in the controls (*p* = 0.03); there was no significant difference in mortality (1.7% DCB vs. 3.6% controls; *p* = 0.53). A randomized trial conducted by Zeller *et al*. [30] found that the one-year patency rate for paclitaxel-coated balloons was lower than that for percutaneous transluminal angioplasty (PTA) in popliteal artery lesions. Specifically, the patency rate for DCB was 17.1%, whereas the patency for PTA was 26.1%. After the 5-year follow-up, the rate of freedom from CD-TLR in the group treated with the DCB was still lower than in the group treated by PTA (70.9% vs. 76.0%) [31]. In another randomized trial conducted by Patel *et al*. [32], comparing paclitaxel, a DCB, with PTA, it was found that after 6 months, the rate of patency in the DCB group was better than in the control group (43% vs. 38%). However, after the one-year follow-up, the survival rate without amputation in the DCB group was significantly lower than in the control group (59% vs. 78%, *p* = 0.01). These findings have raised concerns about the use of DCBs in below-the-knee (BTK) lesions, particularly due to the significant narrowing of arteries after the lumen has been expanded, which poses a challenge to the resistance of the DCBs. Though DCBs have effectively replaced standard balloon angioplasty, post-dilation dissection, elastic recoil, or incomplete lumen expansion due to calcification may still occur [33]. Therefore, concern exists over the failure of balloons to provide calcification lumen with enough short-term mechanical support.

## 3. Overviews of Bioabsorbable Scaffolds in PAD

The initial goal of bioabsorbable stents was to have a device that could offer adequate mechanical support and release an anti-proliferative medication in the short- to medium-term (1–2 years), which is in line with the current popular “leave nothing behind” strategy [10]. Here, the scaffold progressively merges with the lumen during the ensuing years, which lowers the risk of late restenosis and thrombosis brought on by the retention of long-term implants. Following ablation, the stent does not occupy the lumen or cover the collateral branches, providing better patency for future bypass surgery. Furthermore, artifacts produced by implants can be removed during non-invasive procedures [34]. Further evidence has been provided to support the initial objective of these designs (stent ablation, clinical outcomes, and imaging evaluation) [35].

Currently, the ABSORB BVS is the most studied BRS in CAD. Initially, the ABSORB BVS showed promising results. However, in larger randomized trials, the ABSORB BVS led to higher rates of scaffold thrombosis than drug-eluting stents. The ABSORB III trial, a study randomizing 2006 patients, demonstrated a higher risk of adverse events at 5 years, while the risk reached a state of stability within a three-year timeframe [36, 37]. Nowadays, other BRSs with different backbones, such as magnesium, are still being investigated but with caution and in small studies [38].

### 3.1 Mechanisms and Materials of Bioabsorbable Scaffolds Used in PAD

Poly-L-active acid (PLLA) is the primary component most frequently employed in creating bioabsorbable scaffolds, followed by magnesium or iron alloys. PLLA is a semi-crystalline polymer that is broken down into lactic acid when hydrated and enters the citric acid cycle to become carbon dioxide and water before the kidneys and lungs finally eliminate it. The aggregation of macrophages and lymphocytes caused by PLLA throughout the breakdown process results in an inflammatory reaction, especially around the scaffold [39, 40].

**Fig. 1.**
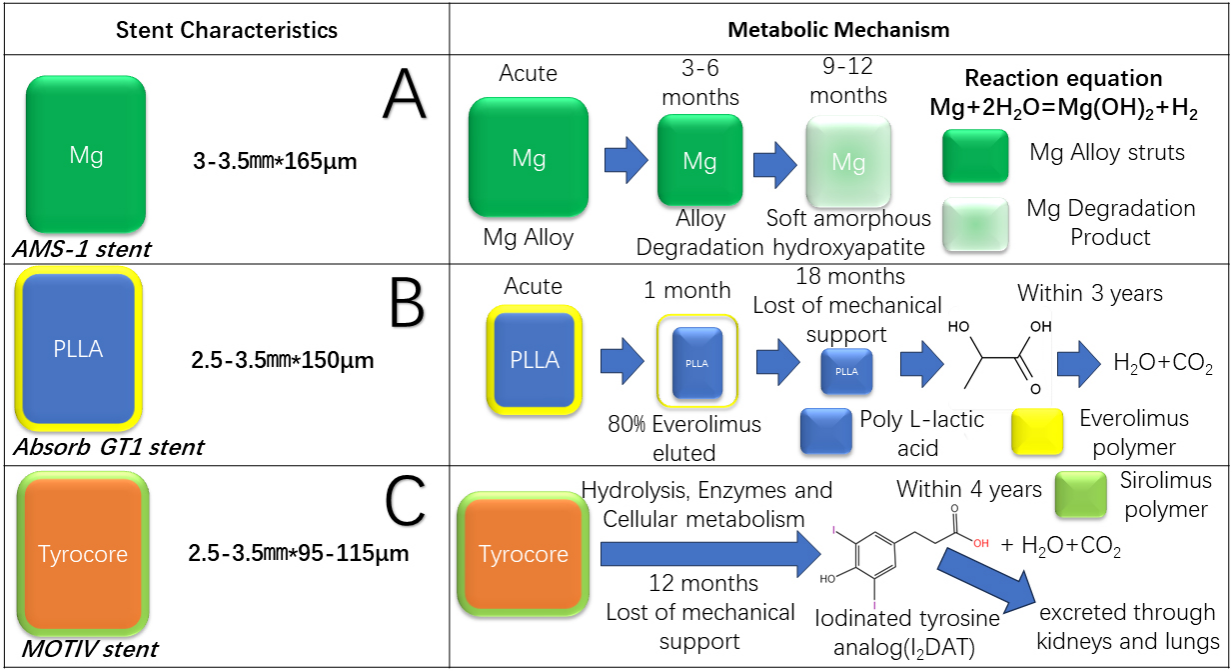
**Mechanisms of three bioresorbable scaffolds**. (Part A) Magnesium alloy stent. (Part B) PLLA stent. (Part C) Tyrocore stent. Part A illustrates the deterioration process of magnesium alloy scaffolds. The picture displays the fundamental reaction equation. Degradation of the magnesium alloy scaffold is initiated within a period of 3–6 months and subsequently transforms into hydroxyapatite, which is absorbed after 9–12 months. Part B illustrates the deterioration of a BVS eluted with everolimus. The release of the drug is often completed within a month, with the stent losing its mechanical reinforcement after approximately a year and a half. Eventually, the stent completely breaks down into water and carbon dioxide through the tricarboxylic acid cycle. Part C illustrates the degradation mechanisms of a Tyrocore BVS, which exhibits accelerated deterioration and experiences mechanical loss within approximately one year. Moreover, the iodinated diphenol, which is a metabolic intermediary, enables the scaffold to be detected and visualized during imaging examinations. AMS, absorbable metal stent; $I_{2}$DAT, iodinated tyrosine analog; PLLA, poly L-lactic acid; BVS, bioresorbable vascular stent.

Compared to modern metallic DESs, however, first-generation PLLA-based BRSs suffer from several significant disadvantages. Since they are not radiopaque, unlike metal stents, it is necessary to designate the proximal and distal ends of the scaffold with tiny markers. Therefore, accurate positioning might be difficult, particularly when overlap is needed. Second, PLLA is harder and more ductile than metals while also possessing lower tensile and mechanical strengths. As a result, even though the PLLA bracket has larger and wider struts, its tensile and radial strengths are still only approximately half those of a metal bracket [41, 42]. Alloy scaffolds are more resistant to scaffold fracture than polymer scaffolds because they have thinner struts, lower contours, and higher radial strengths [43, 44]. Third, the PLLA-made stent is wider at the junction than the metal stent, increasing the surface coverage area of the stent and its degree of adhesion to the vascular endothelium, which causes turbulence and platelet activation [45, 46]. Fourth, certain BRSs have a limited capacity for extension and are vulnerable to breaking if overextended during implantation. Finally, the various PLLA-based BRSs currently on the market must be implanted using a stepwise-balloon-inflation approach, which increases procedural time and the risk of ischemia (Fig. 1) [47].

Given these deficiencies, alloys made of magnesium, iron, and zinc were created as PLLA replacements [48], yet these failed to reduce opacity and pillar thickness (the present AMS (absorbable metal stent)-1, DREAMS 1G, and DREAMS 2G have strut thicknesses ranging from 130 to 165 µm). Early stent failure was caused by the fast reabsorption of these corrosive metals. Although the PLLA-based drug coating technique appears to be the answer, early stent failure still occurs in clinical practice [49]. The most popular alloys are made of magnesium because they offer special benefits. The human body requires the trace metal element magnesium because it plays an important role in enzyme catalysis and cell metabolism [50]. Next to $K^{+}$, ${\mathrm{Mg}}^{2+}$ is the second-most significant cation in cells in terms of both importance and content. It is both biodegradable and has strong biocompatibility [51]. The degrading release of ${\mathrm{Mg}}^{2+}$ from a magnesium alloy scaffold can be low and is non-toxic to humans, given the amount of ${\mathrm{Mg}}^{2+}$ in the human body (0.7–1.0 mmol/L) [52]. Additionally, due to its anti-arrhythmic effects, magnesium has also been used to treat acute myocardial infarction [53, 54]. Moreover, ${\mathrm{Mg}}^{2+}$ can significantly reduce the infarct size, probably because of its resistance to thrombosis and inhibition of microvascular obstruction [55, 56]. The present Magmaris stents are made of magnesium alloys, which comprise a combination of rare earth metals, zirconium (Zr), and yttrium (Y), and lengthen biological absorption by lowering the corrosion rate due to the pure and rapid rate of deterioration *in vivo* (Fig. 1) [57].

Third-generation BRSs are made with Tyrocore, a new polymer mainly composed of an iodinated short-chain polycarbonate copolymer of tyrosine analogs and characterized by a reduction in the release of lactic acid, resulting in less irritation, decreased tissue calcium formation, and improved endothelization, compared to PLLA. The radiopacity of Tyrocore is due to iodine, which is bonded to tyrosine to generate the iodinated diphenol and is visible in imaging without adding markers (Fig. 1) [58].

### 3.2 Device Characteristics

The absorbable metal stent (AMS-1) (Biotronik, Berlin, Germany) is the first absorbable stent system used for BTK revascularization. It was made from a WE43 alloy composed of 93% Mg and 7% rare earth elements. The AMS-1 was a tubular, slotted, balloon-expandable scaffold, which was sculpted by laser from a tube of a bioabsorbable magnesium alloy without drug elution. The mechanical characteristics of the magnesium scaffold were similar to those of stainless-steel stents, including low elastic recoil, high collapse pressure, and minimum amount of shortening after inflation [59]. The AMS-1 system comprises a pre-mounted stent on a quick interchange delivery system. A quick exchange percutaneous transluminal coronary angioplasty (PTCA) catheter serves as the foundation of the delivery system. A balloon located at the distal end of the system can be used to expand the stent. The balloon has two radiopaque markers at either end, while the stent is positioned in the middle of the balloon’s extension between the markers. The diameter and length of the struts are 3 and 3.5 mm and 10, 15, and 20 mm, respectively, while the thickness ranges from 150 to 200 mm (Table 1) [59, 60, 61].

**Table 1.** **Stent characteristics of three bioresorbable scaffolds**.

Characteristics	AMS-1	Absorb GT 1	MOTIV
Scaffold material	Magnesiµm alloy	PLLA	Tyrocore
Drug coating	None	Everolimus+PDLLA	Sirolimus+Tyrocore
Strut thickness	3.0 mm, 165 µm	2.5 mm, 150 µm	2.5 mm, 95 µm
	3.5 mm, 165 µm	3.0 mm, 150 µm	3.0 mm, 105 µm
		3.5 mm, 150 µm	3.5 mm, 115 µm
Crossing profile	1.5 mm	1.44 mm	1.3 mm
Delivery	Single-step inflation	Muti-step inflation	Single-step inflation
Radial strength	0.17 N/mm	0.14 N/mm	0.22 N/mm
Recoil	<8%	2.3%	2.0%
Max expansion over nominal	0.6 mm	0.5 mm	0.75 mm, 2.5–3.0 mm
			0.5 mm, 3.5 mm
Resorption profile	At least 4 months	Loss of mechanical support in 18 months	Vessel uncaged in 12 months
		Resorption in 36 months	Resorption in 48 months

AMS, absorbable metal stent; PLLA, poly L-lactic acid; PDLLA, poly D, L-lactic acid.

A 7 mm poly (D, L-lactide) polymer (PDLLA), coated on a PLLA structure called the Absorb GT1 BRS, regulates the release of the anti-proliferative medication everolimus at a concentration of 100 ${\mathrm{mg/mm}}^{2}$. When ester linkages between the lactide repeat units are hydrolyzed, the lengthy PDLLA and PLLA chains are gradually reduced. Toward the conclusion of the resorption process, particles less than 2 mm in diameter are phagocytosed by macrophages. The components of this device are circumferential hoops that are joined by straight bridges with dual radiopaque platinum markers at either end to assist with fluoroscopic visibility. The scaffold lengths are four-fold (8, 18, 23, and 28 mm), and the Absorb GT1 BVS struts are 157 mm thick. The diameters may be securely post-dilated 0.5 mm beyond their nominal diameter and range from 2.5 to 3.5 mm in thickness (Table 1) [62, 63].

The MOTIV Bioresorbable Scaffold is intended for use in treating BTK disease. MOTIV is composed of Tyrocore and controls the release of the anti-proliferative drug sirolimus at a concentration of 1.97 ug/mm, REVA’s new proprietary polymer, MOTIV is the first bioresorbable scaffold to be licensed for the treatment of BTK disease. There are three sizes of MOTIV. The appropriate strut thicknesses are 95 µm, 105 µm, and 115 µm for lengths of 2.5 mm, 3.0 mm, and 3.5 mm, respectively. The diameters vary from 2.5 to 3.0 mm and may be securely post-dilated 0.75 mm beyond their nominal diameter, while for 3.5 mm, it is 0.5 mm. After implantation, it can offer dependable circulatory support for at least a year before deteriorating over four years (Table 1) [64, 65].

## 4. State-of-the-Art Strategies for BTK Intervention

### 4.1 Implantation Procedures

**Fig. 2.**
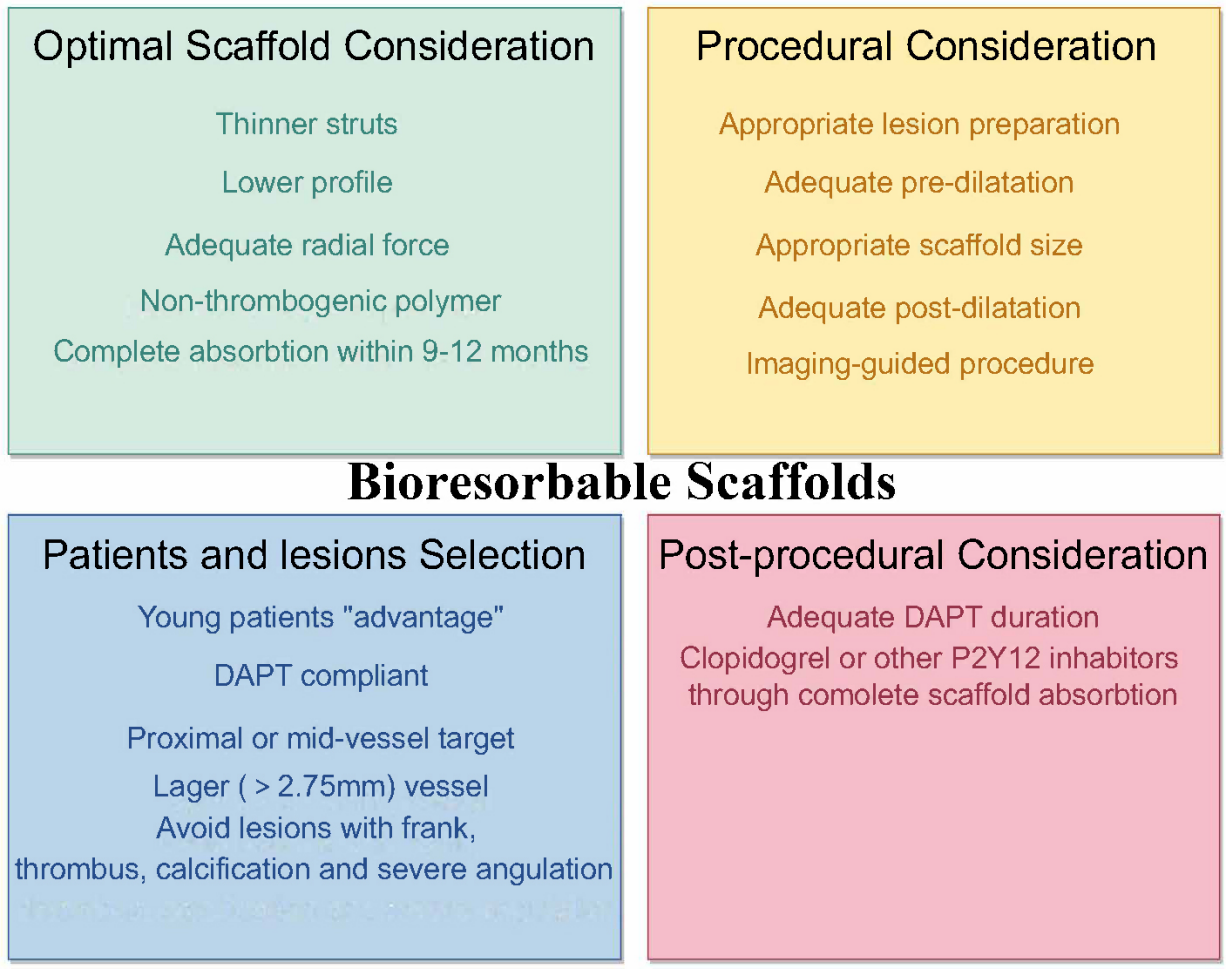
**Risk factors to consider before and following implantation of bioresorbable stents**. Before implantation surgery, the critical factors are choosing suitable stents and ensuring proper lesion management. Following surgery, the most crucial aspect is DAPT. However, there is currently a debate regarding the optimal duration of this therapy and whether routine testing of the *CYP2C19* gene should be conducted to determine the appropriate use of antiplatelet medications. DAPT, dual anti-platelet therapy.

In response to the early practice of BRS in coronary arteries (Absorb II, III), where the lack of mature technical specifications has led to varying degrees of stent stenosis and thrombosis on follow-up [66, 67], the manufacturer and guideline committee worked together to develop corresponding operating specifications and principles [68, 69] (see Fig. 2 and Table 2). Since subsequent studies have rigorously adhered to these guidelines, the outcomes of these studies were improved [70, 71]. Therefore, using BRSs in BTK lesions must first adhere to these guidelines. Currently, we can only implement these guidelines in relation to time.

**Table 2.** **Pre-dilation, vessel sizing, and PSP technical specifications for BRS implantations**.

Technical specifications	Contents
Pre-dilation	Pre-dilated balloon (non-compliant balloon recommended) diameter: reference vessel diameter is approximately 1:1.
	Lesions that cannot be fully dilated require pre-treatment using cutting balloons or rotary milling techniques.
	Placing a BRS is not recommended unless the lesion can be fully dilated.
Proper sizing	Application guidance catheter balloon, online QCA software, and intracavitary imaging technology for guidance.
	Absolute avoidance of bracket selection being too small.
	If the size of the blood vessel is too small (<3.0 mm), it is recommended to use intracavitary imaging techniques to avoid embedding BRS in small blood vessels (<2.75 mm).
Post-dilatation	Using non-compliant balloons.
	The ratio of balloon diameter to reference vessel diameter should be determined based on the specific condition of the lesion.
	Expansion after high pressure (>18 atm).
	Cannot exceed the BRS expansion limit of 0.5 mm (new generation BRS is not limited).

BRS, bioabsorbable scaffold; PSP, pre-dilation, vessel sizing, and post-dilation; QCA, quantitative coronary angiography; 1 atm = 101.325 kPa.

Choosing the right tools and regularly updating the BRS should lessen the negative effects of the stent. Stent research is moving toward smaller struts, higher strengths, and better insertion methods. Although there are concerns that thinner struts may compromise recoil resistance and radial strength, we think that advancements in the polymer can be readily solved. To minimize the burden of excessive thrombosis or calcification load, the BRS should be administered in newly diagnosed patients with extended life expectancies, using diameters and lengths that are compatible with the available BRS size. Moreover, the BRS should only be used to prolong dual anti-platelet treatment (DAPT) in individuals who do not have contraindications to these medications. Based on the three distinct processes involved in the implementation process, a unique technique known as pre-dilation, vessel sizing, and post-dilation (PSP) was created, as shown in Table 2. The PSP method decreases the negative effects of limited stent expansion and weak wall adherence. The findings for first-generation BRSs were equivalent to those for the EESs when using comprehensive PSP, according to the post hoc examination of PSP data from randomized trials. The outcomes are worse than those of other DESs or BMSs when the complete PSP is not used [72, 73]. Finally, reasonable anti-platelet therapy is recommended. We suggest that DAPT be performed for at least 1 year after BRS implantation. If the risk of bleeding is low, it may be prudent to consider using DAPT for at least 2 to 3 years with the current generation of BRSs. A mesh meta-analysis of 64 randomized controlled trials with 102,735 participants revealed that the type of stent appeared to partially influence the probability of adverse events during the follow-up when different DAPT lengths were used. The performance of the BRS appears to be comparable to second-generation DESs in terms of major adverse cardiovascular events (MACEs). However, stent thrombosis (ST) risk appears to rise regardless of the DAPT length.

Even though the use of a BVS in coronary arteries is debatable due to its high rate of restenosis and increased frequency of unfavorable events, the outcomes were attributed to ineffective implantation methods and insufficient strut thicknesses [74]. Thus, the application of these implants in BTK vascular disease has been debated. It is important to note that patients with BTK arterial disease frequently have concomitant conditions, including diabetes and the need for dialysis. The characteristics of infrapopliteal arterial disease are that it frequently involves long-segment chronic total occlusions (CTOs), is diffused, and is extensively calcified. Likewise, the lower extremity vascular bed has a strong impedance for outflow and a relatively moderate flow rate [75]. In addition, small vessel diameters (usually <4 mm) and small outflows are the main characteristics of BTK arteries. The BRS may offer advantages in treating CTO lesions, as the gradual breakdown of a BRS does not compromise the availability of options for secondary interventions and open surgery [76].

### 4.2 Calcification in BTK Arterial Disease

Currently, it is generally accepted that vascular calcification (VC) is an active and complex intracellular molecular process that causes macrophages and vascular smooth muscle cells (VSMCs) to differentiate into osteoclast-like cells by the raising the level of calcium and phosphorus in the blood, VC is a pathologic response to toxic stimuli involving metabolic substances and inflammatory cells [77, 78], for which, intimal and medial VC have both been characterized as subtypes. Atherosclerosis is intimately associated with intimal calcification and results from osteoblast differentiation and apoptosis caused by lipids and inflammatory substances in plaques. Intimal calcification may develop in an attempt to stop the development of aberrant cellular processes, thereby safeguarding the healthy surrounding intima in the process [78, 79]. Although medial calcification does not directly cause luminal stenosis, the resulting decrease in vascular wall elasticity and compliance can ultimately result in recurrent disease. Medial calcification is more common in lower limbs (especially the BTK artery), which is related to the differentiation of smooth muscle cells in the mesothelium [78, 80, 81].

BTK lesions are frequently characterized by severe medial artery calcification (MAC), which makes the arteries stiffer and raises artery pressure. Since MAC is more frequently found in the smaller distal arteries, it is linked to the patient’s poor prognosis, high risk of complications, and high rate of amputation, especially in patients with CLTI [82, 83]. Narula *et al*. [84] discovered that distal small-artery medial calcification was present in 43 of 75 patients (57.3%) and was associated with varying degrees of intimal fibrosis, resulting in mild to severe luminal stenosis. CLTI can result from numerous changes, such as severe intimal hyperplasia, thrombotic occlusions, and CaP deposits caused by MAC [85, 86]. In the amputated limbs of CLTI patients, a strong correlation between MAC in the foot arteries and obstruction of the metatarsal artery was also discovered [87]. MAC also prevents drugs from penetrating the bloodstream, causing postoperative residual stenosis and restenosis. In conclusion, the BTK artery is widely calcified, and the calcification lesions are often lengthy and associated with CTO. This calls into question the role that the BRS plays in revascularizing the BTK arteries. It is currently unknown how calcification influences the outcomes of the BRS implantation since severe calcification has historically been used as an exclusion criterion in most coronary artery BRS trials. Studies have found that the outcomes of coronary artery calcified lesions and non-calcified lesions with BRS implantation may be comparable [88]. In other studies, inserting the BRS for coronary artery CTO lesions that have undergone adequate “lesion preparation” can produce good mid- to long-term effectiveness [89, 90]. The results from coronary artery studies seem to suggest that a BRS can make a difference in the “encirclement” of BTK calcification if the “lesion preparation” can be improved as much as possible.

However, it must be remembered that “lesion preparation” is based on damage and that striving for perfection increases problems; if it is too cautious, the treatment impact of long-term calcification lesions will not be favorable. Notably, POBA, specialized balloons (cutting balloons, scoring balloons, chocolate balloons, serration balloons), intravascular lithotripsy, and atherectomy are some of the various “lesion preparation” techniques [91].

### 4.3 Thin Blood Vessels in BTK Arterial Disease

Lesions were not accurately screened in the Absorb series of tests, especially in the early studies. Since many tiny coronary arteries, with a diameter of less than 2.5 mm, were included in the research (nearly 20% in Absorb III), the risk of thrombosis in thick and broad struts was dramatically increased [92, 93, 94]. However, the BTK artery has a relatively small diameter. Most BTK arteries have a diameter of less than 4 mm in various patterns [95]. The native diameter of BTK arteries is unfavorable due to age and significant calcification. Therefore, it appears that the BRSs may be compromised by thrombus development in the constricted lumens of BTK arteries, with unfavorable results. Thus, thinner struts are still required to solve this problem.

## 5. Bioabsorbable Scaffolds in PAD

Over the past 20 years, due to the active intervention of BRSs in coronary artery stenosis, researchers have also made preliminary findings on the effectiveness and safety of BRSs in BTK arteries (Table 3, Ref. [61, 63, 96, 97, 98, 99, 100, 101, 102, 103, 104, 105, 106, 107]).

**Table 3.** **Studies evaluating the mid- to long-term performance of bioresorbable scaffolds in below-the-knee arterial disease**.

Trial (year)	Study design	Drug coating	Lesion length, mm	Limbs (n)	Lesions (n)	Primary patency, %				Limb salvage, %			
						3 m	6 m	1 y	3 y	3 m	6 m	1 y	3 y
Bosiers (2005) [61, 96]	Prospective case series	Magnesium alloy	11 (2–20)	20	20	89.5	-	73.3	-	100.0	-	94.7	-
Bosiers (2009) [97]	Prospective case series	Magnesium alloy	10.6 ± 4.9	59	72	-	31.8	-	-	93.2	87.6	-	-
Stabile (2016) [99]	Retrospective registry	Biolimus	23.5 ± 9.4	30	-			93.4		-	-	96.7	-
Varcoe (2016) [63, 98]	Prospective case series	Everolimus	19.2 (5–50)	38	43	-	96.0	96.0	87.3	-	100.0	100.0	100.0
Dia (2019) [100]	Retrospective case series	Everolimus	30.9 (10–60)	31	-	-	-	96.7	-	-	-	96.8	-
Parikh (2019) [106]	Prospective case series	Sirolimus	≤56	30	-	-	-	88.9	-	-	-	-	-
Kum (2019) [101]	Retrospective case series	Everolimus	22.7 ± 17.2	41	53	-	95.0	86.0	-	-	93.0	85.0	-
Huizing (2021) [102]	Pooled analysis	Everolimus	21 (15–30)	121	161		97.3	91.7	86.6 (2 y)	-	-	-	-
Varcoe (2023) [103]	Prospective case series	Everolimus	43.8 ± 31.8	173	179	-	-	79.7	-	-	98.8	97.7	-
Bosiers (2023) [104, 105]	Prospective case series	Sirolimus	29.5 (5–100)	60	-	-	90	88.3	-	-	97.0	95.0	-
Brodmann (2023) [107]	Prospective case series	Sirolimus	31.9 ± 13.9	30	31	-	83.3	-	-	-	100	-	-

n, the number of people; m, month; y, year. Lesion length is represented in two ways: either as the mean value ± standard deviation (mean ± SD) or as the median value with the 25th percentile subtracted from the 75th percentile (mean (Q25–Q75)).

### 5.1 Current Clinical Evidence of Bioabsorbable Scaffolds in BTK Artery Disease

#### 5.1.1 Metal Alloy Bare BVS 

Peeters and colleagues placed a total of 23 absorbable metal stents (AMS) (Biotronik, Berlin, Germany) in 20 patients without using drug elution technology [61]. Imaging at the 3-month follow-up revealed a primary clinical patency of 89.5% (one patient died in a non-surgical related event). No major or minor amputations were required in any of the patients, and the average improvement in Rutherford class was 2.3 at the 3-month assessment. However, due to limitations in the follow-up and the number of participants, this experiment can only be considered a preliminary study [61]. Bosiers *et al*. [96] subsequently presented the findings from the 12-month follow-up, revealing that the survival rate, primary patency rate, and limb salvage rate for the patients were 85.0%, 73.3%, and 94.7%, respectively. A randomized scientific control was completed by Bosiers *et al*. [97] to compare the efficacy of AMS and stand-alone periodic PTA in the BTK lesions. The results of the 6-month follow-up were disappointing, with an angiographic patency rate for lesions treated with AMS (31.8%) significantly lower (*p* = 0.013) than the rate for those treated with PTA (58.0%). Although angiography was not used to evaluate the results, this study overshadowed the effectiveness of AMS without drug elution in maintaining the BTK lumen. The findings suggest that magnesium alloy absorbable stents exhibit favorable safety profiles in managing below-the-knee artery disease. However, their long-term patency rate is inferior to that of PTA.

Ferroalloys possess distinctive biodegradability, favorable biocompatibility, and exceptional mechanical qualities. In terms of radial support [108], ferroalloy BVS surpasses magnesium alloy BVS, rendering it more appropriate for calcified blocked arteries [109]. The efficacy of ferroalloy scaffolds in animal tests is exceptional. In 2018, Qi *et al*. [110] published findings from their laboratory research on BVSs made from iron and polylactic acid. They also conducted animal experiments by implanting the BVS in the abdominal aorta of New Zealand white rabbits. These experiments confirmed that the stent has exceptional mechanical qualities. The material can undergo total degradation within a period of 3–6 months, and there were no notable instances of endothelial hyperplasia or inflammatory reaction observed 12 months post-surgery. In 2020, Lin and colleagues [111] published a study on the outcomes of using sirolimus-coated, galvanized iron alloy stents containing 0.05% nitrogen in animal coronary arteries. The stents showed a favorable degradation rate and biocompatibility.

The findings from the zinc alloy stent experiment revealed that despite its inadequate mechanical strength, it did not significantly impact the efficacy of animal trials [112]. However, further animal experiments and clinical investigations are required to validate its potential applications.

The available research on absorbable alloy bare brackets supports the conclusion that magnesium alloy can offer safe short-term benefits; however, its long-term impacts are unsatisfactory. Iron and zinc alloys are primarily utilized in animal investigations, whereas the status of their application in randomized controlled human trials remains uncertain.

#### 5.1.2 Everolimus BVS 

Varcoe *et al*. [63] conducted a single-arm study of the ABSORB BVS in predominantly simple BTK lesions in 33 patients. They noted freedom from a clinically driven target vessel revascularization rate of 96% at 12 months, with a 100% technical success rate and excellent procedural safety. A continuous 3-year follow-up showed that the patient’s primary patency rate was 81%, the freedom from CD-TLR was 87%, and the limb salvage rate was 100% [98]. After 5 years, the patient’s primary patency rate was 72%, the freedom from CD-TLR was similar to the 3-year results, and the limb salvage rate was unknown [113]. Data from the study by Varcoe are undoubtedly encouraging; from the long-term results, there was no significant difference in limb salvage and patency rates compared to most DES, proving the enormous potential of BRSs. However, since it forms the current study with the largest number of patients included, with only about 100, it is difficult to avoid the possibility of selection bias. Moreover, the inclusion criteria almost do not involve long-term lesions (over 20 mm), which raises doubts about the clinical application of BRSs.

The treatment of BTK lesions with a single-arm BRS has also been the subject of several retrospective investigations by groups, including Stabile, Dia, and Kum [99, 100, 101]. These studies only involved a limited number of patients, only possessed 1-year follow-up findings, and did not include controls or randomization; the incidence of CD-TLR was 6.7%, 4.9%, and 7%, respectively, while the primary patency rate over one year was 93%, 96%, and 86%. In addition, Dia *et al*. [114] published 2-year follow-up results: 49 BRSs were implanted in 41 arteries of 31 patients with a median age of 67 years and most suffering from severe infrapopliteal disease, with 49% of the lesions being chronic thrombotic occlusion. There was no perioperative bleeding or stent thrombosis. All patients had successful surgical outcomes, and 93.5% of the patients were spared from clinically driven target vessel failure at two years. The main patency percentage was 87.1% after two years, and every patient remained alive.

The 2021 pooled analysis conducted by Huizing *et al*. [102] examined 121 individuals with below-the-knee arterial disease treated with everolimus bioabsorbable stents. The analysis was based on data from a database and yielded the following findings: Restenosis was observed in 21 scaffolds during a period of 24 months, leading to an overall patency rate of 91.7% and 86.6% at 12 and 24 months, respectively. Six scaffolds underwent target lesion revascularization, resulting in an independence of 97.2% from clinically driven target lesion revascularization at 12 months and 96.6% at 24 months. After 30 days, a single patient had to undergo an amputation because of worsening tissue damage. There was a total of 18 fatalities within 24 months. The 24-month overall survival rate was 85%.

Varcoe *et al*. [103] conducted a LIEF BTK trial from 2020 to 2022 to examine the effects of an everolimus-eluting BVS compared to angioplasty in 261 patients with below-the-knee arterial disease. The patients were allocated to the two treatment groups in a 2:1 ratio, respectively. In the one-year follow-up, it was found that 74% of the patients in the BVS group successfully reached the therapeutic endpoint, which was freedom from occlusion of target blood vessels, above the ankle joint amputation, CD-TLR, and binary restenosis of target lesions. In comparison, only 44% of the patients in the angioplasty group achieved this endpoint (95% confidence interval, 15 to 46; one side *p* < 0.001 for superiority). Five patients in the BVS group did not reach the safe endpoint, which was defined as freedom from major adverse limb events at 6 months and peri-procedural death. However, all individuals in the angioplasty group achieved this safe endpoint [103].

The above data suggests that everolimus BVSs are effective in treating below-the-knee arterial disease and are not worse than PTA. However, some studies have noted that everolimus BVSs had a comparable rate of repeat revascularization at the 1-year follow-up, compared to everolimus DESs, despite showing poor performance in coronary intervention mid-term angiography. Nevertheless, individuals undergoing treatment with BVSs face a heightened susceptibility to subacute and late stent thrombosis. Despite the absence of comparative data on PAD, the findings from coronary treatment indicate the necessity to provide additional evidence regarding the long-term benefits of everolimus BVSs [115, 116].

#### 5.1.3 Sirolimus BVS 

As previously reported, REVA was used to create a Fantom sirolimus-eluting bioabsorbable stent for coronary artery disease and a new generation of tyrosine-derived complex Tyrocore. During the follow-up period of 6 to 9 months, over 250 patients had BRS implants. The long-term quantitative flow ratio (QFR) analysis that followed demonstrated the stent’s improvement in ischemia [117, 118]. Then, the same material was used for sirolimus-eluting MOTIV stents for BTK vascular patency. In 58 patients, the average lesions were 29.46 mm long, with 47% of them being calcified, and 76 BRS were implanted. An initial patency rate of 90%, a CD-TLR rate of 3%, and a limb salvage rate of 97% were recorded during the 6-month follow-up. The primary patency rate was 88.3%, the CD-TLR rate was 3%, and the limb salvage rate was 95% after the one-year follow-up [104, 105].

The Credence BTK BVS (manufactured by Meril Life Science in Vapi, Gujarat, India) is a stent that releases sirolimus and is composed of PLLA. A preliminary trial involving 30 participants (FIM study, CTRI/2016/11/007473) demonstrated a primary patency rate of 88.9% after 1 year. At 30 days, 6 months, and 12 months, the advantages of the Credence BTK were noteworthy, as there were notable enhancements in the Rutherford grading and ankle-brachial index observed in all patients [106], despite the intended completion date of the experiment being in the July of this year, we encountered difficulty in locating the relevant 3–5-year follow-up data.

During the VIVA 2023 conference, Brodmann presented the findings of a study on the effectiveness of R3 Vascular Magnitude sirolimus-eluting bioabsorbable stents in treating symptomatic BTK arterial disease. The study evaluated the 6-month outcomes of this treatment. Thirty patients (with a total of 31 lesions) were enrolled and categorized into three groups according to the Rutherford grading system, which consisted of four to six levels. Six months following surgery, the patency rate was 83.3%, and the limb salvage rate was 100%. Additionally, the postoperative minimum lumen diameter benefit was measured to be 2.2 ± 0.4 mm, indicating an improvement in the diameter of the blood vessel after surgery [107]. Ongoing trials such as IBS Titan (sirolimus-eluting BVS) and PTA control trials on below-the-knee arterial lesions in the United States (NCT05971394) and China (NCT04849325) are actively enrolling patients and are anticipated to yield robust results.

Sirolimus BVSs, such as the everolimus BVS, hold significant potential. However, it is premature to draw definitive conclusions due to the scarcity of available evidence.

**Fig. 3.**
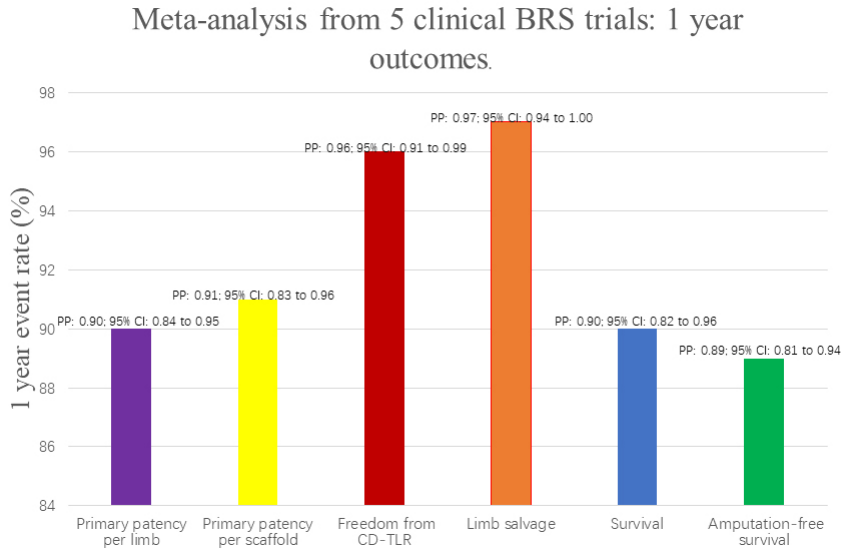
**Summary of 1-year pooled results from a meta-analysis of five clinical studies**. BRS, bioresorbable scaffold; CI, confidence interval; CD-TLR, clinical-driven target lesion revascularization; PP, proportion.

A meta-analysis of five medium-grade BRS procedures for BTK lesions included 155 patients and 160 treated limbs, and the results showed a combined 12-month patency rate of 90%, a CD-TLR free rate of 96%, limb salvage rate of 97%, patient survival rate of 90%, and amputation free survival rate of 89%. This meta-analysis demonstrates that BVSs have good 12-month patency and clinical outcomes for BTK arterial disease, even in individuals with numerous and complicated lesions (Fig. 3) [119].

### 5.2 Other Clinical Evidence of Bioabsorbable Scaffolds in PAD

As indicated in Section 5.1, in the intervention of BTK arterial disease, magnesium alloy BVSs have comparable safety to PTA yet lack long-term effectiveness. Additional alloy BVSs are currently undergoing animal experimentation. On the other hand, the everolimus BVS has demonstrated notable short-term effectiveness, although concerns have been raised about its safety during mid-term follow-ups. There is a scarcity of data available for sirolimus BVSs, although a control experiment is presently in progress.

We have also discovered clinical evidence of BVSs in cases of femoropopliteal arterial disease. Clinical studies were conducted in the GAIA study to assess the efficacy of IGAKI-TAMAI in individuals suffering from occlusive superficial femoral arterial (SFA) disease. The binary restenosis rates of the IGAKI-TAMAI stent were 39.3% and 67.9% after 6 and 12 months of follow-up, respectively. The target lesion revascularization (TLR) rate was 25.0% after 6 months and increased to 57.1% after 12 months. The rate of secondary patients after 1 year was 89.3% following CD-TLR [120]. The REMEDY stent is composed of semicrystalline PLLA, a commonly utilized bioresorbable polymer that has been scientifically established as safe for medicinal applications. Bontinck *et al*. [121] performed an observational study on 99 patients to assess the effectiveness of REMEDY stents in treating SFA disease. The percentage of TLR rose from 19% after 6 months to 33% after 12 months. The initial rate of blood vessel openness was 68% after 6 months and 58% after 12 months. The incidence of continued openness of the secondary blood vessel was 85% at 6 months and 86% after 12 months. After 12 months, two individuals underwent surgical procedures to have their limbs amputated. A Japanese study has also investigated the effectiveness of REMEDY stents in the SFA. The primary patency percentage after 12 months was 88.6%, which was below the predetermined criteria. There was no discernible disparity in the degree of diameter stenosis at 9 to 12 months. Throughout the observation period, there were no instances of mortality, significant limb removals, or distal embolisms associated with using instruments or surgical procedures. Over 5 years, the ankle-brachial index (ABI) demonstrated sustained and notable enhancement compared to the initial measurement. The occurrence rates of TLR, MACEs, and significant adverse events affecting the cardiovascular system and limbs at 12 months were 95.8%, 91.7%, and 87.5%, respectively. At 5 years, these rates were 85.4%, 72.1%, and 62.5%, respectively [122]. A clinical trial (n = 32) on using ESPRIT BVSs to treat symptomatic claudication in external iliac and femoropopliteal occlusive vascular disease (ESPIRIT 1) assessed the use of everolimus BVSs in the above-the-knee arterial vessel. Of the treated lesions, 89% were in the femoropopliteal artery. The binary restenosis rates at 1 and 2 years were 12.1% and 16.1%, respectively, while the TLR rates were 8.8% and 11.8%, respectively. There were no security concerns associated with the gadget or program [123]. There is ongoing enrollment in a trial called Efemoral, which is a single-arm, open-label study. This experiment aims to examine the use of sirolimus-coated scaffolds in individuals with symptomatic peripheral vascular disease caused by stenosis or occlusion of the femoropopliteal artery. The trial is registered with the identifier NCT04584632.

## 6. Conclusions

Two major limitations are associated with the currently used biodegradable scaffolds: they have less radial strength and a higher strut thickness. As a result, there is increased platelet activation, leading to thrombosis and intimal hyperplasia compared to DESs. Although the addition of drug elution technology inhibits endothelial growth, its overall effect is still unclear. Research and development efforts have mainly focused on improving the strength and thickness of the scaffolds. The emergence of Tyrocore demonstrates that bioengineering research can enable organic polymers to achieve, and even exceed, the strength of corrosive metals. DCBs comply with the principle of “leave nothing behind”, yet they do not provide sufficient increases in the lumen diameter. DESs offer complete support and anti-proliferative effects, but the remaining stent body continuously affects the newly formed intima in blood vessels, leading to suboptimal treatment outcomes. BRSs aim to combine the benefits of both DCBs and DESs. However, the current clinical evidence suggests that the performance of BRSs is similar to DESs. Although we have made progress, we have not yet achieved the desired results. Nonetheless, the search for an ideal device remains important and should continue.

## Abbreviations

ABI, ankle-brachial index; AMS, absorbable metal stent; BMS, bare metal stent; BRS, bioresorbable scaffold; BTK, below-the-knee; BVS, bioresorbable vascular stent; CAD, cardiovascular disease; CD-TLR, clinically driven target region recurrence; CLTL, chronic limb-threatening ischemia; CTO, chronic total occlusion; DAPT, dual anti-platelet therapy; DCB, drug-coating balloon; DES, drug-eluting stent; $I_{2}$DAT, iodinated tyrosine analog; MAC, medial artery calcification; MACE, major adverse cardiovascular event; MI, myocardial infarction; PAD, peripheral arterial disease; PDLLA, poly (D, L-lactide); PLLA, poly (L-lactic acid); POBA, plain old balloon angioplasty; PSP, pre-dilation, vessel sizing, and post-dilation; PTA, periodic translational angioplasty; PTCA, percutaneous transluminal coronary angioplasty; QCA, quantitative coronary angiography; QFR, quantitative flow ratio; SFA, superficial femoral arterial; ST, stent thrombosis; TLR, target lesion revascularization; VC, vascular calcification; VSMCs, vascular smooth muscle cells.
